# Carbonization of Polydopamine-Coating Layers on Boron Nitride for Thermal Conductivity Enhancement in Hybrid Polyvinyl Alcohol (PVA) Composites

**DOI:** 10.3390/polym12061410

**Published:** 2020-06-24

**Authors:** Youjin Kim, Jooheon Kim

**Affiliations:** School of Chemical Engineering and Materials Science, Chung-Ang University, Seoul 156-756, Korea; becon272727@gmail.com

**Keywords:** boron nitride (BN) 1, polymer–matrix composites (PMCs) 2, heat treatment 3, polydopamine coating, thermal properties

## Abstract

Inspired by mussel adhesion proteins, boron nitride (BN) particles coated with homogeneous polydopamine (BNPDA) were prepared, and through an annealing process, a carbonized PDA layer on the surface of BN was obtained, which exhibited a nanocrystalline graphite-like structure. The effect of carbonization of PDA coating layer on BN particles was characterized by various analytical techniques including SEM, TEM, Raman spectroscopy, and XPS. When the resulting particles were used as a thermally conductive filler for polyvinyl alcohol (PVA) composite films, enhanced thermal conductivity was observed compared to raw BN composite due to the ordered structure and improved solubility in water. Furthermore, the homogeneous dispersion of the filler and excellent flexibility of the modified composite film with 21 wt % filler may be attributed to compatibility with the PVA chain. As the whole fabrication process did not use toxic chemicals (mainly water was used as the solvent), it may contribute to green and sustainable chemistry.

## 1. Introduction

Polydopamine (PDA) coating inspired by mussel adhesive proteins has attracted wide interest because of its simple fabrication process and excellent coating ability on almost all types of substrates [1]. PDA is prepared by the self-polymerization of dopamine (DA) in an alkaline aqueous solution (pH 8.5) [2]. The mechanism of polymerization involves the oxidation of the catechol group of DA, which becomes 5,6-dihydroxyindole. Intermolecular cross-linking through further oxidation results in cyclized DA monomers and yields a PDA layer on the substrate surface [3,4,5]. Due to the strong and universal adhesion properties, PDA has been used to coat various substrates including boron nitride (BN) nanotube, graphene, copper nanowire, Al_2_O_3_, and graphene oxide [6,7,8,9,10]. In addition, the conversion of the PDA layer to a carbonized graphite-like nanostructure through thermal annealing in an inert atmosphere has been reported [11]. For example, Ryu et al. [12] developed a carbon nanotube fiber coated with carbonized PDA having high mechanical strength and electrical conductivity up to 5000 Scm^−1^. Li et al. [13] used carbonized PDA as a thermoelectric material, taking advantage of the high electrical conductivity due to the atomic arrangement from an amorphous structure to an ordered structure. Ma et al. [14] also reported the enhanced mechanical strength and conductivity of graphene-based fibers with carbonized PDA coating.

Due to rapid growth in modern electronic devices with miniaturization and high-density integration, thermally conductive polymer composites have attracted increasing attention due to their potential in addressing heat dissipation problems [15,16]. Moreover, advances in flexible electronics, such as flexible displays, wearable sensors, and electronic skins, requires compatible thermal interface materials (TIM) exhibiting high flexibility while maintaining high thermal conductivity. To overcome the large amount of heat generated during the operation of electronic devices, polymer-based composites are generally used because of their lightweight, low cost, and easy processability [17]. To address the intrinsic low thermal conductivity (TC) of polymers, highly thermally conductive materials, such as carbonaceous and ceramic materials, have been incorporated into polymer matrices. Among them, BN, known as “white graphene”, has been extensively investigated considering its chemical stability and outstanding TC [18]. However, the highly inert surface and absence of functional groups result in poor affinity with polymers [19]. In addition, the hydrophobicity of BN causes low dispersibility during the composite fabrication process, especially when the solvent is water. 

In this study, we modified the surface of BN with PDA via the self-polymerization of DA followed by heat treatment at various temperatures for PDA carbonization. The morphology and transition of the chemical structure of heat-treated BN coated with PDA (BNPDA) particles were characterized by electron microscopy (SEM, TEM), Raman spectroscopy, and X-ray photoelectron spectroscopy. In addition, the resulting carbonized PDA was used as a thermally conductive filler infiltrated with polyvinyl alcohol (PVA). PVA was chosen as the polymer matrix in the present study due to its water solubility, superior mechanical properties including flexibility, and abundant hydrogen bonds, which are compatible with the catechol group of PDA [20]. The TC of PVA composite films, filled with BN coated with carbonized PDA (carbonized BNPDA) at various temperatures, was examined and compared with that of pristine BNPDA and raw BN composite films. 

## 2. Experimental

### 2.1. Materials

Hexagonal boron nitride (h-BN) (12 μm) was obtained from Momentive Performance Materials (Waterford, NY, US). DA hydrochloride and PVA (MW 31,000~50,000) were obtained from Sigma-Aldrich (St. Louise, MO, USA). Tris(hydroxymethyl)aminomethane (Tris) (99.8%) was purchased from Alfa Aesar (Haverhill, MA, USA).

### 2.2. Preparation of BNPDA

A 1 L round bottom flask was charged with raw BN (2 g), DA (400 mg), Tris (10 mM), water (700 mL), and ethanol (300 mL). The pH value of the mixture was then adjusted to 8.5 by adding hydrochloric acid and stirred for 24 h at room temperature. After the reaction, the dispersion was centrifuged at 10,000 rpm for 30 min, followed by vacuum filtration and washing with DI water several times to remove impurities and self-polymerized PDA. After drying in a convection oven at 50 °C overnight, BNPDA was prepared. The BNPDA particles were heated to 400 (BNPDA-400), 800 (BNPDA-800), 1000 (BNPDA-1000) °C in a tube furnace for 1 h in an Ar atmosphere (heating rate = 5 °C/min) to obtain a carbonized PDA-coated surface.

### 2.3. Fabrication of BNPDA/PVA Composite Film

Various concentrations of aqueous BNPDA (18 mL) were obtained by sonication for 30 min. PVA powder (3.2 g) was then dissolved in the as-prepared solution by stirring at 90 °C for 2 h. This mixture was cooled down to 40 °C until an adequate viscosity was obtained and poured into a polytetrafluoroethylene (PTFE) mold. The doctor blade technique was used to produce films with a uniform thickness, which were dried at 40 °C for 12 h. Finally, the PVA composites were hot-pressed at 90 °C to obtain a flat surface.

### 2.4. Characterization

#### 2.4.1. Electron Microscopy

The morphology of BNPDA samples and cross-sectional fracture image of composite films were characterized by field emission scanning electron microscopy (FE-SEM; Sigma, Carl Zeiss, Oberkochen, Germany). For the cross-sectional image, composite films were immersed in liquid nitrogen and then fractured. The morphology of BNPDA samples, before and after carbonization, was also examined by field emission transmission electron microscopy (FE-TEM; JEM-F200, JEOL Ltd., Akishima, Japan) with energy-dispersive X-ray spectrometry (EDS).

#### 2.4.2. Raman Spectroscopy

Structural analysis of carbonized PDA was performed by Raman spectroscopy (DXR2xi; Thermo Fisher Scientific, Waltham, MA, USA) with laser excitation at 532 nm.

#### 2.4.3. X-Ray Photoelectron Spectroscopy (XPS)

X-ray photoelectron spectroscopy (XPS, K-alpha+; Thermo Fisher Scientific, Waltham, USA) was conducted to evaluate the chemical bonding states of carbonized PDA. 

#### 2.4.4. X-Ray Diffraction (XRD)

X-ray diffraction (XRD, New D8-Advance; Bruker-AXS, Billerica, MA, US) patterns were obtained with Cu-Kα radiation (0.154056 nm). The XRD measurement was conducted over a 2θ range of 10–60°.

#### 2.4.5. UV–Vis Spectroscopy

Absorbance of the film was measured by UV–Vis spectroscopy (V-760; JASCO, Hachioji, Japan) in the wavelength from 350 nm to 800 nm.

#### 2.4.6. Thermal Properties

Thermal properties were analyzed by measuring thermal diffusivity using the laser flash method (LFA, NanoFlash LFA 447; Netzsch Instruments Co., Selb, Germany) at room temperature. Thermal conductivity value was calculated by multiplying the thermal diffusivity, density, and specific heat capacity of the composite films.

## 3. Results and Discussion

BNPDA particles were synthesized by the self-polymerization of DA at an alkaline pH based on previously reported methods [14,21]. The optimized ratio between BN and DA was set to 1:0.2; when the input amount of DA exceeded that ratio, excess DA self-polymerized into spherically shaped PDA and adhered to the BNPDA surface (Figure S1, Supporting Information). During the coating process, water and ethanol (ratio of 7:3) were used as the solvent considering the hydrophobicity of BN; as the polymerization progressed, the color of the solution changed from light brown to black (Figure S2). After centrifugation and vacuum filtration of the resulting solution, uniformly coated BNPDA was obtained. To carbonize PDA, which is adhered to the BN surface, each sample was thermally treated at 400, 800, and 1000 °C for 1 h in an Ar atmosphere. 

**Figure 1:**
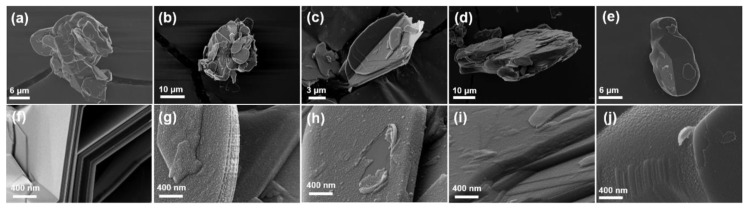
FE-SEM images of (**a**,**f**) raw boron nitride (BN), (**b**,**g**) heat-treated BN coated with PDA (polydopamine) (BNPDA), (**c**,**h**) BNPDA particles heated to 400 °C (BNPDA-400), (**d**,**i**) BNPDA particles heated to 800 °C (BNPDA-800), (**e**,**j**) BNPDA particles heated to 1000 °C (BNPDA-1000).

Figure 1 shows the morphology of raw BN, BNPDA, and carbonized BNPDA annealed at different temperatures. There was no structural deformation after heat treatment for the low magnification image (Figure 1a–e). As shown in Figure 1g, the magnified image of BNPDA revealed that the BN surface was evenly covered by a PDA layer without cracks or inconsistencies and had a rough surface compared to smooth raw BN surface (Figure 1f). The high magnification image revealed that BNPDA annealed at 400 °C still had a rough surface, and the area peeled off by external force had a smooth surface of pristine BN, indicating the presence of the PDA layer (Figure 1h). As shown in Figure 1i,j, after high temperature annealing (above 800 °C), the surface became relatively smooth because of the carbonization of the PDA layer into a graphite-like nanostructure [11]. 

**Figure 2:**
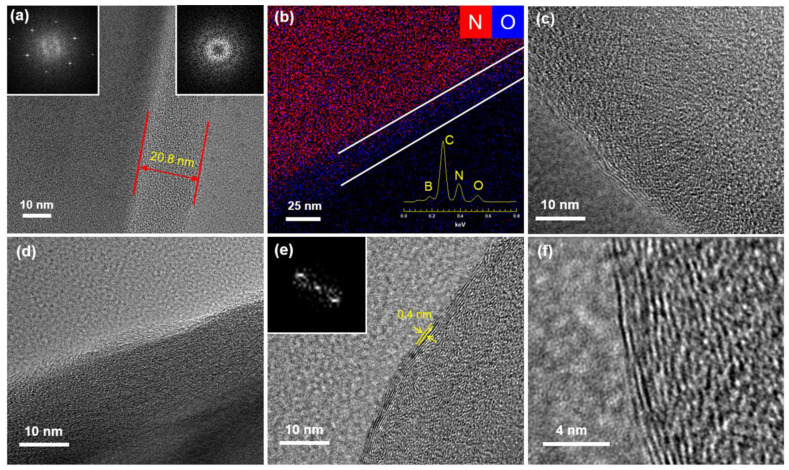
FE-TEM images of edge of (**a**) BNPDA, (**b**) EDS atomic analysis of BNPDA, FE-TEM images of edge of (**c**) BNPDA-400, (**d**) BNPDA-800, (**e**,**f**) BNPDA-1000.

The carbonization of PDA on the surface of BN after heat treatment was further characterized by TEM and EDS. Figure 2a shows the edge of particles coated with 20.8 nm-thick PDA. The selected area electron diffraction (SAED) pattern of the inside of the particle exhibited a typical six-fold symmetry due to the crystalline structure of hexagonal BN [22]. However, the SAED pattern of the edge where only the PDA layer exists revealed a diffuse ring pattern, indicating the amorphous structure of PDA. The EDS analysis of BNPDA particle is shown in Figure 2b. As demonstrated by the EDS spectrum of BNPDA (inset graph in Figure 2b), PDA consists of carbon and oxygen from the catechol group and nitrogen from the amine group. The EDS overlay mapping image revealed the uniform presence of oxygen on BNPDA. On the other hand, nitrogen exhibited a relatively weak signal at the edge of the particle compared with the strong signal of nitrogen from BN in the inner part. Figure 2e,f shows the TEM image of the edge of BNPDA after annealing at 1000 °C for 1 h in an Ar atmosphere. In contrast to the amorphous phase of the PDA layer before the annealing process, a number of stacking layers were observed after heat treatment, which indicated the carbonization of the PDA layer into a nanocrystalline graphite-like structure [11]. Moreover, these staking layers were getting clearer with the increasing of annealing temperature (Figure 2c–e). The interlayer spacing of carbonized PDA was around 0.4 nm, which is consistent with previous findings [12,23]. The measured layer distance of carbonized PDA was greater than the typical distance of graphite layers (0.3 nm); based on a hypothetical structure of carbonized PDA [24], we hypothesized that graphitic and pyridinic N could weaken the aromatic conjugated π–π stacking, causing a repulsive force between layers and increasing d spacing.

**Figure 3:**
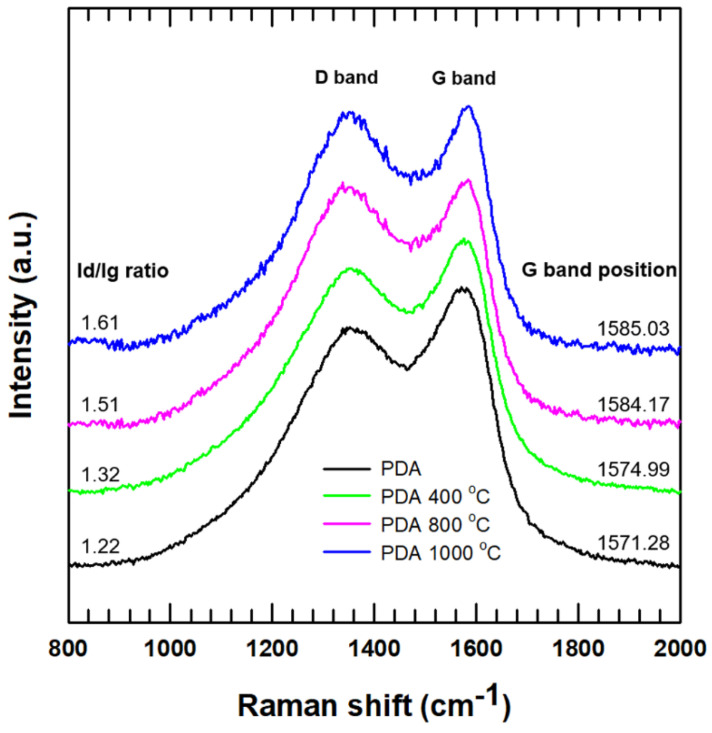
Raman spectrum of carbonized PDA with increasing annealing temperatures.

Figure 3 shows the Raman spectroscopy of PDA and heat-treated PDA at various temperatures. All of the samples had two distinct peaks including a D band at ~1350 cm^−1^ and a G band at ~1580 cm^−1^. The area integral intensity ratio of two peaks (Id/Ig ratio) was increased, and the peak position of the G band tended to shift to a higher Raman shift with increasing annealing temperature. The increased Id/Ig ratio and gradual blue shift indicated that the PDA layer, which consists of amorphous carbon, was converted to a nanocrystalline graphite-like structure [13,25].

**Figure 4:**
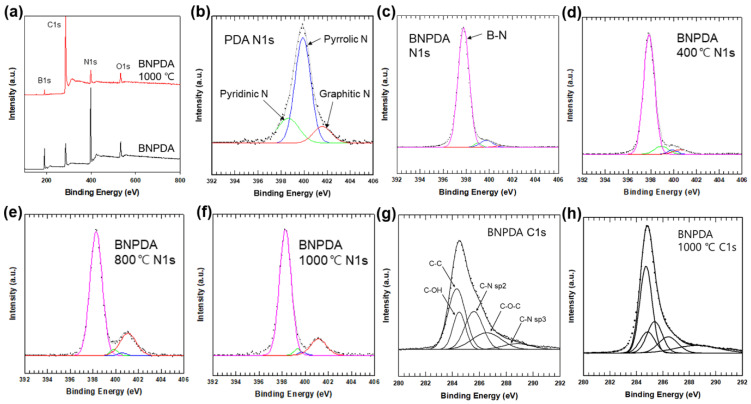
(**a**) XPS survey spectra of BNPDA and BNPDA-1000. N1s XPS spectra of (**b**) PDA, (**c**) BNPDA, (**d**) BNPDA-400, (**e**) BNPDA-800, and (**f**) BNPDA-1000. C1s XPS spectra of (**g**) BNPDA and (**h**) BNPDA-1000.

XPS analysis was conducted to verify the extent of the carbonization of PDA on the surface of BN. As XPS can measure the elemental composition of a sample up to 10 nm deep, it is useful for investigating chemical transition on the surface in our study. The wide survey spectra of BNPDA and carbonized BNPDA heat-treated at 1000 °C are shown in Figure 4a, which revealed the presence of oxygen, boron, carbon, and nitrogen. 

Table 1 shows the surface atomic percent of BNPDA and carbonized BNPDA at different temperatures. With increasing annealing temperature, the carbon at% was increased significantly, whereas the boron and nitrogen at% was decreased. The similar decreasing trend of boron and nitrogen and increasing carbon suggest that that the surface of BN was gradually covered by carbonized PDA with increasing annealing temperature. Figure 4b shows the N1s spectra of PDA, which could be deconvoluted into three peaks positioned at 398.6, 399.9, and 401.6 eV, corresponding to pyridinic N, pyrrolic N, and graphitic N, respectively. In the N1s spectra of BNPDA (Figure 4c), a strong peak at 397.8 eV was additionally observed due to N–B bonds from BN. The N1s spectra of BNPDA according to the annealing temperature are shown in Figure 4d–f. As expected, the peak corresponding to graphitic N was increased with increasing annealing temperature. Moreover, the C1s spectra of BNPDA before and after annealing at 1000 °C (Figure 4g,h) were deconvoluted into five components: C–C (284.3 eV), C–OH (284.5 eV), C–N sp2 (285.6 eV), C–O–C (286.5 eV), and C–N sp3 (288.8 eV) [12,14]. After heat treatment, the intensity of the peaks assigned to C–C and C–N sp2 was increased, which corresponds well with the hypothetical structure of carbonized PDA [23]. 

**Figure 5:**
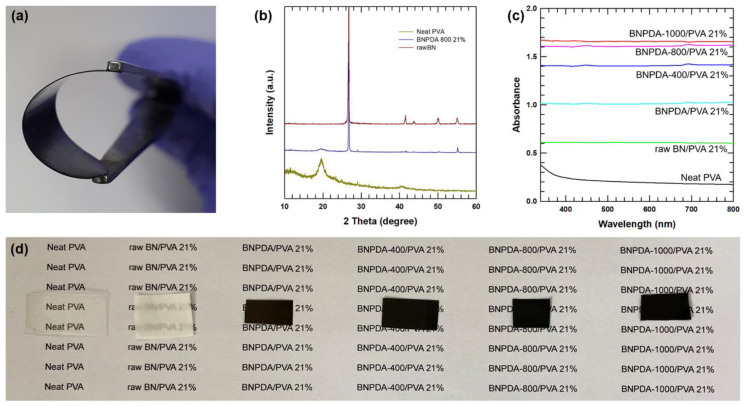
(**a**) Digital photograph of flexible BNPDA-800/PVA composite film. (**b**) XRD pattern of raw BN, BNPDA-800/PVA, and neat PVA. (**c**) UV–Vis absorbance spectra of composite films and (**d**) their optical images.

Based on the results of characterization, PDA on the surface of BN was successfully carbonized after heat treatment. To investigate the role of carbonized BNPDA as a thermally conductive filler, composite films based on PVA were prepared according to the annealing temperature of BNPDA. Figure 5a shows the digital image of the as-prepared 21 wt % carbonized BNPDA/PVA film. Hydrophilicity induced by PDA coating resulted in the homogeneous dispersion of BNPDA in the PVA matrix, allowing the BNPDA/PVA composite to retain the original strength and flexibility of neat PVA (Figure 5a). The crystalline structure of carbonized BNPDA/PVA composite film was investigated by XRD analysis. As shown in Figure 5b, characteristic peaks can be detected at 26° and 55°, which originated from the (002) and (004) planes of BN, respectively. However, other characteristics peaks from BN with 2θ values of 41.6, 43.8, and 50.1 corresponding to the (100), (101), and (102) planes were missing or barely detected. This could be attributed to the amorphous PDA layer on the BN surface [6]. Even after the carbonization of PDA, both the nanocrystalline and amorphous parts are present on the coating layer [11]. In the case of pure PVA, peaks can be detected at 19.6° and 29.1°, which may be attributed to the intense intermolecular interface linking of the PVA chains and the hexagonal ordering inside the polymer [26]. As expected, these two peaks can be detected in the XRD pattern of the carbonized BNPDA/PVA composite film. Figure 5c shows that neat PVA exhibited low absorbance compared to other composite films, showing high transparency in the visible wavelength region (Figure 5d). Increased absorbance was measured with increasing annealing temperature of the composite films, and this could be attributed to the nanocrystalline graphite-like structure at the edge part of BNPDA after carbonization. Homogeneous crystalline structure is beneficial for absorption of UV–Vis light [27].

**Figure 6:**
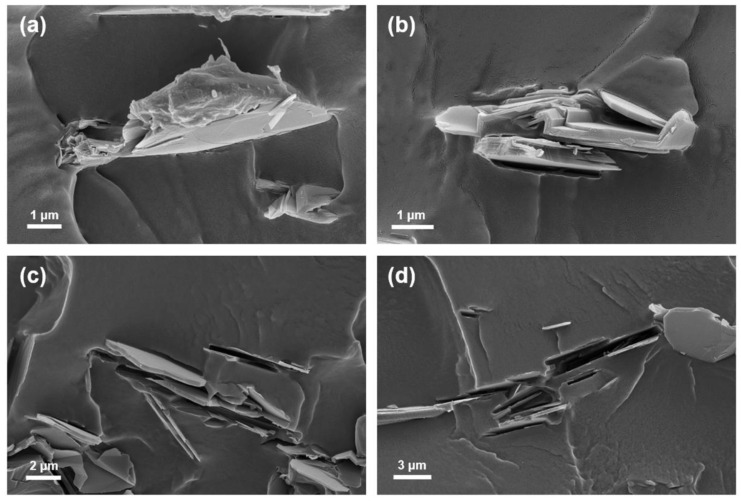
Cross-sectional FE-SEM images of PVA composite films filled with (**a**) BNPDA, (**b**) BNPDA-400, (**c**) BNPDA-800, and (**d**) BNPDA-1000.

As shown in Figure 6, the cross-sectional fracture morphology of the BNPDA/PVA composite film with respect to annealing temperatures was evaluated by FE-SEM. Figure 6a shows BNPDA embedded into the PVA matrix, demonstrating good affinity with each other. The –NH and –OH groups from PDA could form strong hydrogen bonds with –OH from PVA chains, resulting in the gluing effect [28]. With the increasing annealing temperature of BNPDA, the hydrogen bonds between the filler and matrix could be broken through the carbonization process, leading to a decrease in interface affinity. The cross-sectional image of the BNPDA-1000/PVA composite film revealed that there was an empty space where the BNPDA particle had been located, indicating poor interactions between the filler and matrix (Figure 6d). 

**Figure 7:**
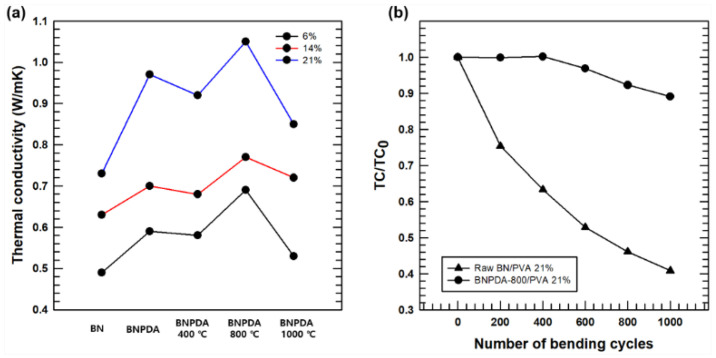
(**a**) Thermal conductivity of PVA composite films with raw BN and BNPDA. (**b**) The ratio of thermal conductivity before and after flexibility test.

Figure 7a shows the M-shaped TC graphs of PVA composite films including raw BN/PVA and BNPDA/PVA with different annealing temperatures. After PDA coating, a sharp enhancement in TC was observed compared with that of the raw BN composite without PDA coating. A similar result was reported by Shen’s group. [21], who indicated that increased TC may be attributed to the improved dispersibility in the PVA matrix and the enhanced affinity between the filler and matrix via hydrogen bonds. We investigated the TC change of PVA composites according to the extent of carbonization and annealing temperature. The composite film with filler annealed at 400 °C showed decreased TC compared with the TC of the film annealed at 0 °C, which may result from the destruction of hydrogen bonds during the carbonization process. Notably, with all three filler fractions (6, 14, and 21 wt %), the TC was increased when the annealing temperature was set to 800 °C. This could be attributed to the progression of PDA carbonization on the surface of BN and increased dispersity of carbonized BNPDA in the PVA matrix. Heat is mainly carried by phonons in non-metallic fillers [29], and the heat propagates more efficiently in crystals than amorphous materials [30]. Carbonization of BNPDA induced by the annealing process increased the crystallinity of the PDA coating layer with a higher annealing temperature, which leads to more lattice vibration of the phonon flow [31,32].

Accordingly, with a filler fraction of 21 wt %, the TC of BNPDA-800/PVA was as high as 1.05 W/mK, which was 44.1% higher than that of raw BN/PVA. However, the TC of BNPDA-1000/PVA was lower than that of BNPDA-800/PVA despite the enhanced intrinsic TC after carbonization under higher annealing temperature; this was because of poor interfacial affinity, which can cause phonon scattering. This result is in good agreement with the result of cross-sectional fracture morphology analysis. The Flexibility test was conducted with raw BN/PVA 21%, and BNPDA-800/PVA 21%, respectively, by measuring the TC ratio as a function of bending cycling. The results are shown in Figure 7b. The composite filled with raw BN showed decreased TC with the bending cycles, indicating the poor affinity between PVA matrix and filler because of the inert surface of BN. Compared to the raw BN film, the TC of the film filled with BNPDA-800 (21%) stays constant after 400 bending cycles, and relatively maintains its high TC even after 1000 bending cycles. Therefore, BNPDA-800/PVA composite is more suitable for the needs for flexible electronic devices than the raw BN/PVA film.

## 4. Conclusions

In summary, we prepared carbonized BNPDA by thermal annealing in an Ar atmosphere. The carbonization of PDA on the surface of BN enhanced not only the thermal properties by converting the amorphous carbon layer to a nanocrystalline graphite-like structure but also the hydrophilicity of BN, resulting in increased dispersibility when used as a filler for PVA composite films. The BNPDA/PVA composite film filled with 21 wt % of BNPDA carbonized at 800 °C exhibited enhanced TC compared with the TC of the raw BN/PVA composite. However, when the annealing temperature exceeded 800 °C, its TC was lower owing to the absence of hydrogen bonding between the filler and matrix after carbonization, which can cause strong phonon scattering. In the future, with additional surface modifications to increase the affinity between the filler and matrix, the TC of composite films may be improved using carbonized BNPDA.

## Figures and Tables

**Table 1:** Quantitative comparison of elements detected from XPS spectra (At%).

Sample	Atomic Ratio (%)			
	O1s	B1s	C1s	N1s
BNPDA	7.15	33.45	24.33	35.07
BNPDA-400	9.14	25.44	37.88	27.54
BNPDA-800	6.26	7.46	76.33	9.95
BNPDA-1000	5.06	7.7	78.55	8.7

## Supplementary Materials

The following are available online at https://www.mdpi.com/2073-4360/12/6/1410/s1, Figure S1: FE-SEM images of BNPDA with various input ratio of BN and DA, Figure S2: Digital photograph images showing color change of BNPDA solution after 24 h reaction.
