# Supplementary material for: Carbonization of Polydopamine-Coating Layers on Boron Nitride for Thermal Conductivity Enhancement in Hybrid Polyvinyl Alcohol (PVA) Composites
Source: Polymers (Basel). 2020 Jun 24;12(6):1410. doi: 10.3390/polym12061410 (PMC7361685; doi:10.3390/polym12061410)
Supplement: Supplementary file 1 [file polymers-12-01410-s001.pdf]

Supplementary material

Carbonization of Polydopamine-Coating Layers on Boron Nitride for Thermal Conductivity Enhancement in Hybrid Polyvinyl Alcohol (PVA) Composites

Youjin Kim, Jooheon Kim*

School of Chemical Engineering and Materials Science, Chung-Ang University,

Seoul 156-756, Korea

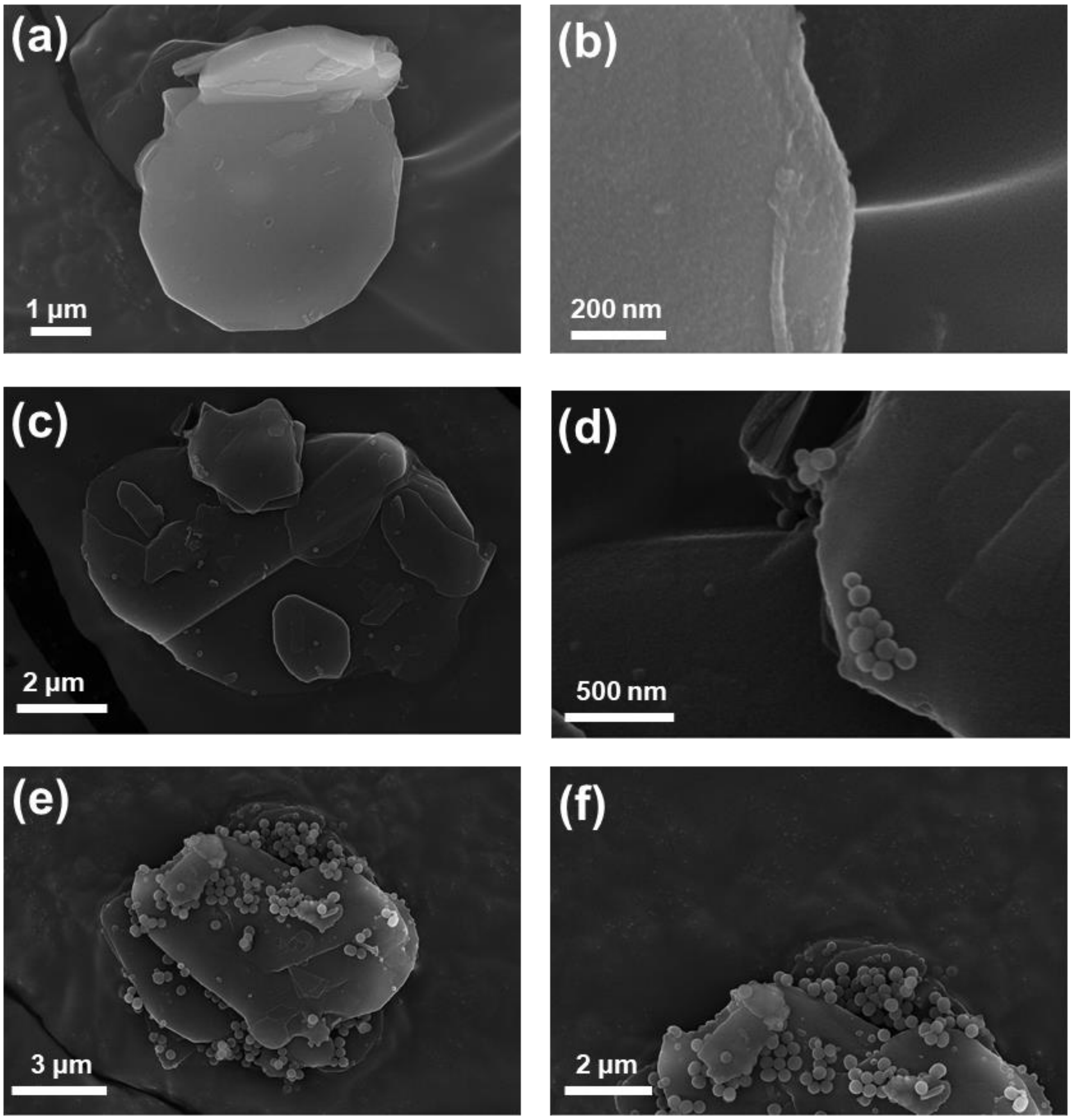

Figure S1. FE-SEM images of BNPDA with various input ratio of BN and DA (a, b) 1:0.2, (c, d) 1:0.4, and (e, f) 1:0.8.

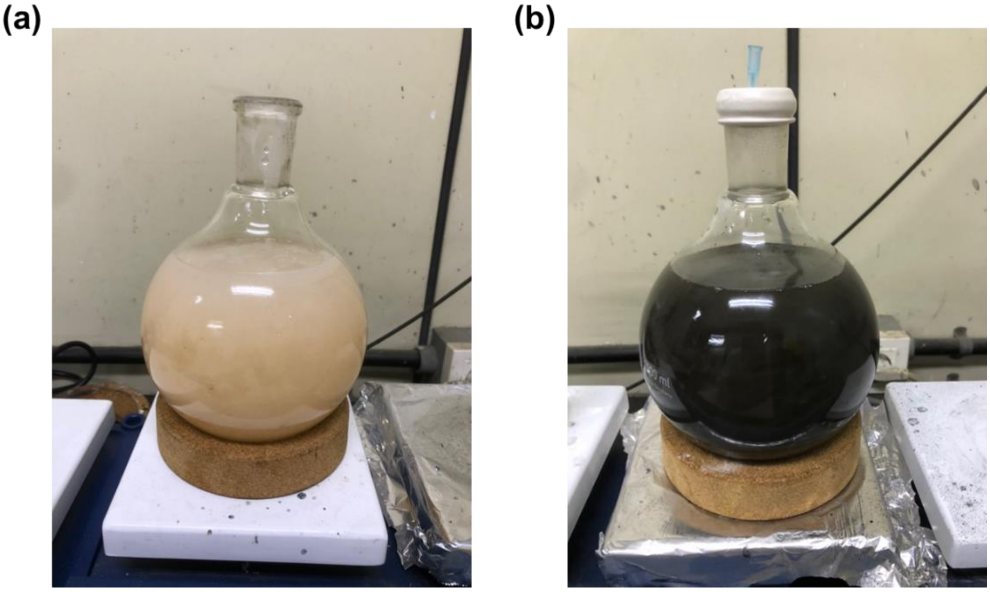

Figure S2. Digital photograph images showing color change of BNPDA solution after 24 h reaction.
